# Psychological aspects of patients with intestinal stoma: integrative review^1^

**DOI:** 10.1590/1518-8345.2231.2950

**Published:** 2017-12-11

**Authors:** Natália Michelato Silva, Manoel Antônio dos Santos, Sara Rodrigues Rosado, Cristina Maria Galvão, Helena Megumi Sonobe

**Affiliations:** 2 MSc, Doctoral student, Escola de Enfermagem de Ribeirão Preto, Universidade de São Paulo, PAHO/WHO Collaborating Centre for Nursing Research Development, Brazil, Doctoral Scholarship in Programa Enfermagem Fundamental.; 3 Free-Lecturer, Associate Professor, Faculdade de Filosofia Ciências e Letras, Universidade de São Paulo, Ribeirão Preto, SP, Brazil.; 4 MSc, Doctoral student, Escola de Enfermagem de Ribeirão Preto, Universidade de São Paulo, PAHO/WHO Collaborating Centre for Nursing Research Development, Brazil, Doctoral Scholarship in Programa Enfermagem Fundamental.; 5 Full Professor, Escola de Enfermagem de Ribeirão Preto, Universidade de São Paulo, PAHO/WHO Collaborating Centre for Nursing Research Development, Brazil.; 6 PhD, Professor, Escola de Enfermagem de Ribeirão Preto, Universidade de São Paulo, PAHO/WHO Collaborating Centre for Nursing Research Development, Brazil

**Keywords:** Ostomy, Digestive System, Perioperative Care, Psychological Adaptation, Rehabitlitation, Review

## Abstract

**Objective:**

to analyze evidences of psychological aspects of patients with intestinal stoma.

**Method:**

integrative review with search of primary studies in the PsycINFO, PubMed, CINAHL and WOS databases and in the SciELO periodicals portal. Inclusion criteria were: primary studies published in a ten-year period, in Portuguese, Spanish or English, available in full length and addressing the theme of the review.

**Results:**

after analytical reading, 27 primary studies were selected and results pointed out the need to approach patients before surgery to prevent the complications, anxieties and fears generated by the stoma. The national and international scientific production on the experience of stomized patients in the perioperative moments is scarce.

**Conclusion:**

it is recomendable that health professionals invest in research on interventions aimed at the main psychological demands of stomized patients in the perioperative period, respecting their autonomy on the decisions to be made regarding their health/illness state and treatments.

## Introduction

The increase in the prevalence of Colorectal Cancer (CRC) along with Inflammatory Bowel Diseases (IBD) implies the need to establish approaches that integrate the psychological aspects resulting from such pathologies to the other dimensions of the health care of this population. Both diseases are characterized as potentially disabling chronic conditions and present the same risk and prevention factors^1^.

As factors for the prevention of damages related to the abovementioned diseases, there is physical activity and consumption of dietary fiber-rich foods, that is, those of plant origin such as fruits, vegetables and whole grains. Risk factors include consumption of red meat, processed meats, alcoholic beverages, smoking, body fat and abdominal fat. Family history of CRC, genetic predisposition to development of chronic intestinal diseases and age are also factors that influence the increase in the incidence and mortality of this cancer^2^.

Surgical intervention is a modality of treatment indicated both for CRC and for IBD and may result in the need for intestinal ostomy. This procedure consists in the temporary or definitive deviation of the colonic effluent, with exteriorization either of the ileum (ileostomy) or the colon (colostomy). As a result of this surgery, it is necessary to use faecal collecting equipment^2^. 

Patient have to face the challenge of acquiring skills to live with the altered body and experience a psychosocial transition. The use of collecting equipment is associated with negative feelings, such as fear, anguish, sadness and helplessness, which can prompt self-deprecating experiences, linked to feelings of mutilation, loss of health and self-esteem, and reduced self-efficacy and a sense of chronic uselessness and incapacitation, among other emotions. Stoma patients experience changes in their lives especially related to their social network (work and leisure) and to sexuality, aggravating their feelings of insecurity and fear of rejection^3^.

The possible negative psychological outcomes and emotional issues arising from the stoma make it essential the provision of comprehensive patient care, with an interdisciplinary and specialized approach to the needs of patients and their families, with a view to full physical, emotional and social recovery towards rehabilitation. It is necessary to prepare patients, mainly during the perioperative period, when they experience anxiety and distress before the unknown - the “stoma”. This preparation must include pre-operative education, demarcation of the stoma and guidance on self-care for patients and their families, in the postoperative period, as well as the referral to the Assistance Program for Stoma patients of the Unified Health System (SUS).

The assistance program aims to offers specialized professional support to patients outside the hospital environment, helping them in the transition from hospital to home care. Different strategies are used to assist them in developing skills for self-care and the required materials are provided. Therefore, it is imperative that the interdisciplinary team know the sociocultural and clinical characteristics of this clientele so so as to carry out proper planning and implementation of effective strategies for this approach^4^.

Individuals with chronic colorectal disease need psychological support and, in some cases, psychotherapeutic intervention with the objective to provide a space to work on aspects that may contribute to coping with the disease, through constant monitoring and guidance^5^.

In view of the complexity of the theme and the intense emotional experiences brought about by the need to live with a limiting condition, this study has the objective to analyze the evidence on the psychological aspects of patients with intestinal stoma.

## Method

Integrative Review (IR) is the appropriate method to reach the proposed goal of analyzing and synthesizing research in a systematized way, to contribute to decision making, deepening the theme and improving clinical practice^6^.

To carry out the IR, the following steps were followed: identification of the theme and selection of the research question; establishment of criteria for inclusion and exclusion of studies; categorization of studies; evaluation of studies included in the integrative review; interpretation of results and synthesis of the main results evidenced in the analysis of the included articles^6^.

The guiding question of the present integrative review was: what is the recent scientific production on psychological aspects of patients with intestinal stoma?

The Scientific Electronic Library Online (SciELO) periodicals portal and the following databases, considered important in the context of health and available online, were used to search for primary studies: American Psychological Association (PsycINFO), National Library of Medicine National Institutes of Health (PubMed), Cumulative Index to Nursing and Allied Health Literature (CINAHL) and Web of Science (WOS).

The search was carried out in June 2017, concomitantly, in the periodicals portal and in the three databases, using controlled descriptors (specific vocabulary of each database). Thus, in the SciELO periodicals portal and in the CINAHL database, the following descriptors were used: *psychological and colostomy*; on the PsycINFO: *psychological adjustment/emotional adjustment and colostomy*; on the PubMed: *psychological (adaptation psychological)* and *colostomy*; and on the WOS: *psychological and colostomy and surgery*. These descriptors were combined, using the Boolean operator “and”, until the studies corresponding to the delimited inclusion and exclusion criteria were obtained.

Inclusion criteria were: primary studies, published between 2006 and 2016, in Portuguese, English and Spanish ​​and available in full length. Exclusion criteria were: literature reviews, secondary studies (e.g., systematic reviews), letters, editorials, case reports, case studies, and primary studies whose participants were children and/or adolescents.

The selection of the primary studies was carried out by two reviewers with experience in the activity, and the results were then compared for the delimitation of the review sample. For the extraction of information from the studies included in the review, an instrument with the following items was used: identification of the study, methodological characteristics and methodological rigor assessment^7^.

The primary studies were classified according to the Level of Evidence (LE). In the classification, the author considers that, there is a hierarchy of evidences that depends on the clinical question of the study, ; in the case of the clinical question of Intervention/Treatment or Diagnosis/Test, the strength of the evidence is classified into seven levels (level I - stronger: evidence from systematic review or meta-analysis of all relevant randomized controlled trials). When the clinical question is of Prognosis/Prediction or Etiology, the authors propose the classification of the strength of the evidence into six levels (level I - evidence from synthesis of cohort or case-control studies). In the case of a clinical question about Meaning, the strength of the evidence is classified into six levels (level I - evidences from meta-synthesis of qualitative studies)^8^.

The descriptive form for the analysis of the evidenced results was adopted, in which the synthesis of each study included in the review was presented, as well as comparisons between the researches.

## Results

**Figure 1:**
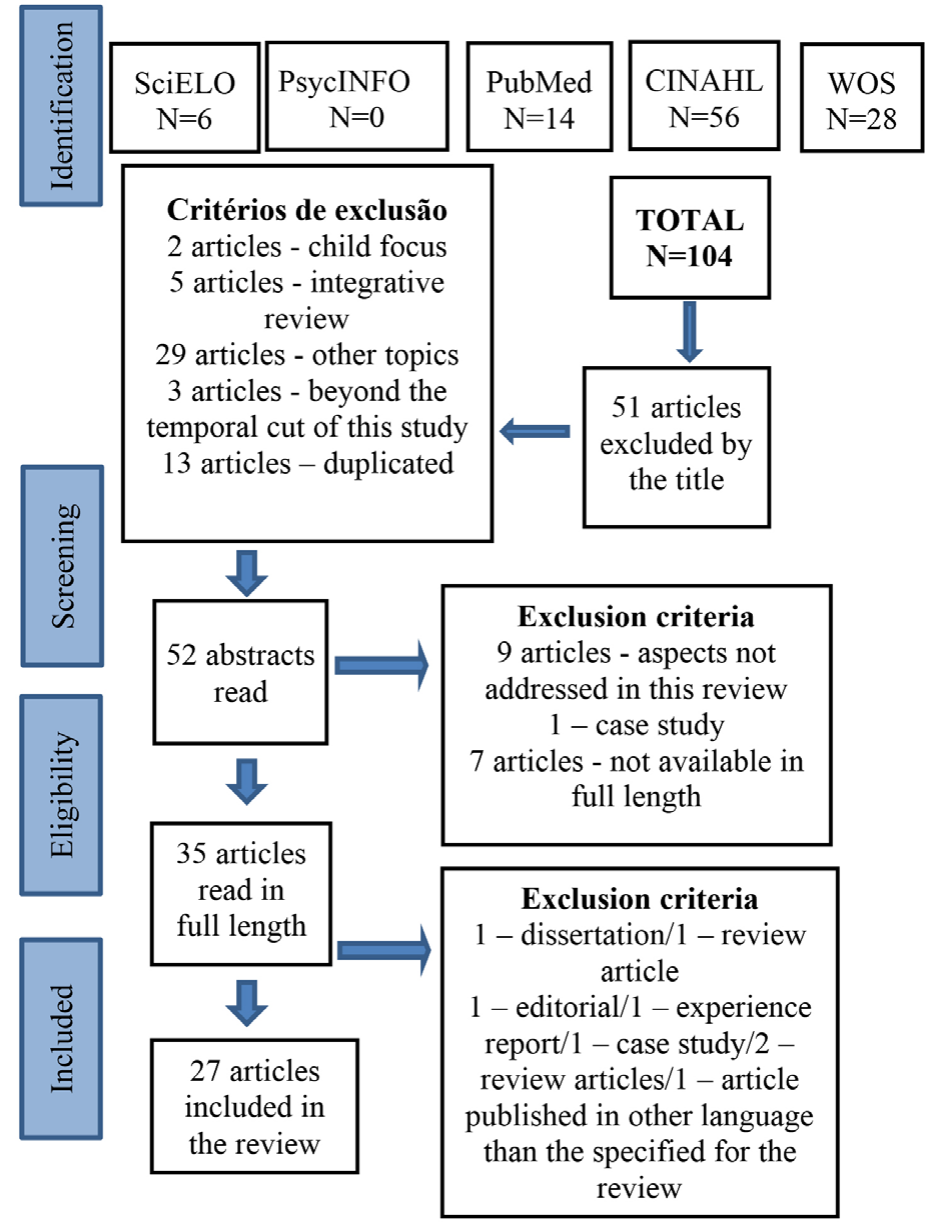
Adaptation of the *Flow Diagram* of the selection process of articles of the integrative review^9^, according to the *Preferred Reporting Items for Systematic Reviews and Meta-Analyses* (PRISMA)

We identified, preliminarily, 107 records in the search in the selected databases and in the periodicals portal. After reading the titles, 51 articles were excluded, as two articles focused on children, five articles were integrative reviews, 29 articles did not address the topic studied, three articles did not match the time cut established for the review, and 13 articles were duplicated. After this stage, the abstracts of 52 articles were read, of which 17 articles were excluded, because nine did not address the theme of the review, one was a case study and seven were not available in full length. After reading 35 articles in full length, six were excluded, for one was a dissertation, one was a review article, one was an editorial, one was an experience report, one was a case study, two were review articles and one article was published in French, which was not a language ​​specified for this review. Thus, 27 primary studies comprised the sample of the present IR. The selection of the primary studies was performed according to the flowchart described in Figure 1.

Regarding the primary studies, four studies were carried out in Brazil, six in China, two in Spain, two in Turkey, two in Sweden and one in each of the following countries: Cuba, Taiwan, Australia, Ireland, Scotland, England, Bosnia and Herzegovina, the United States and Germany, with 22 studies published in English, two in Portuguese and three in Spanish. As for the authors’ home institution, they were all linked to universities. Regarding the year of publication, one study was published in 2006, one in 2008, two in 2007, three in 2009, four in 2010, two in 2011, three in 2012, three in 2013, four in 2014 and four in 2016 .

**Figure 2:**
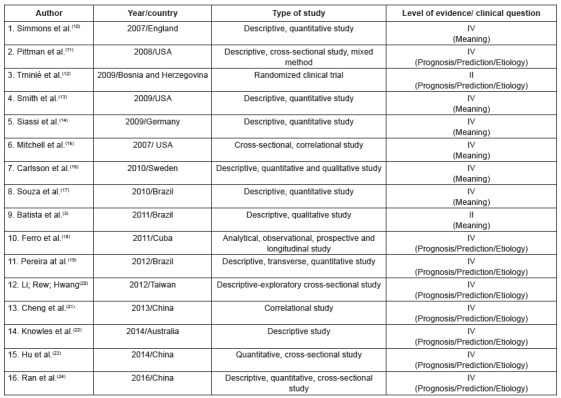
Characteristics of the primary studies grouped in the category “factors associated with adjustment in the transition to a life with colostomy”. Ribeirão Preto, São Paulo, Brazil, 2017

**Figure 3:**
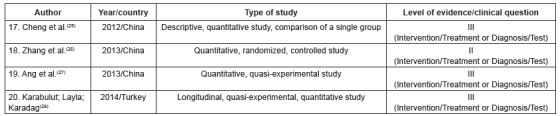
Characteristics of the primary studies grouped in the category “effects of different intervention strategies for optimization of psychosocial adjustment”. Ribeirão Preto, São Paulo, Brazil, 2017

**Figure 4:**
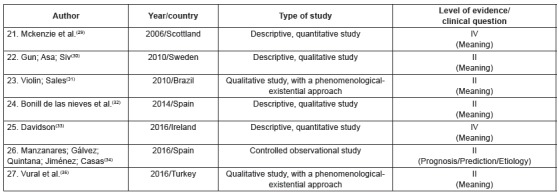
Characteristics of the primary studies grouped in the category “understanding the subjective experience of illness/becoming ill”. Ribeirão Preto, São Paulo, Brazil, 2017

The primary studies were grouped into three categories of analysis, according to their thematic similarity, namely: 1. “factors associated with adjustment in the transition to a life with colostomy” (n = 16), 2. “effects of different intervention strategies for optimization of psychosocial adjustment” (n = 4), 3. “understanding of the subjective experience of illness/becoming ill” (n = 7). Figures 2, 3 and 4 present the characteristics of the primary studies included in the review, according to each delimited category.

The studies analyzed in the integrative review were categorized into three thematic axes: “factors associated with adjustment in the transition to a life with colostomy”, “effects of different intervention strategies to optimize psychosocial adjustment” and “ understanding of the subjective experience of illness/becoming ill”.

Eight primary articles were linked to the thematic axis “factors associated with adjustment in the transition to a life with colostomy”, as described below.

A study developed in England evaluated the acceptance of the stoma and the social interaction in 51 individuals, providing strong evidence of self-efficacy in care with the stoma, acceptance of the condition, interpersonal relationship, and the location of the stoma with adaptation. The study concluded that there is a need to address psychosocial concerns, focusing on negative thoughts and encouraging social interactions^10^.

A study of 239 patients with intestinal stoma in the United States described important relationships between demographic factors (age, marital status, income) and clinical status (type of stoma), with complications that affected the quality of life. These results reinforce the need for individualized care in what regards biopsychosocial aspects^11^.

In another study from Bosnia and Herzegovina, the quality of life of three groups of patients undergoing CRC surgery, one group with colostomy, one group without colostomy and one control group were also evaluated. The control group presented better physical, cognitive and social functioning compared to CRC patients, besides a lower frequency of diarrhea and constipation. The emotional functioning and body image of the colostomy group were unfavorable in relation to the control group, and among CRC patients, those with stoma had worse results, suggesting that psychology should integrate the therapeutic plan^12^.

In order to assess the quality of life related to adaptation to the collecting equipment, a study in the United States compared two groups, one with definitive stoma and the other with a temporary one. There was overall satisfaction and the quality of life increased over time in the case of patients with permanent stoma. Temporality of the stoma may interfere with adaptation, but there is the paradoxical situation in which people who are physically better are worse in the inner aspects^13^.

In another study from Germany, with 79 patients submitted to colorectal surgery, the correlation of quality of life with personality aspects was evaluated and personality was found to exert a strong and lasting effect on the quality of life after surgery, highlighting the influence of clinical variables^14^.

In another study developed in the United States, demographic and clinical variables, and quality of life were identified to be related to embarrassment in the case of people living with a colostomy bag and the study concluded that the feeling of embarrassment may negatively impact the person’s quality of life^15^.

Another study carried out in Sweden evaluated the quality of life related to preoperative concerns for the creation of a stoma and the first six postoperative months in 57 patients. There was a lower quality of life among participants in the first postoperative month compared to the sixth month, due to the adaptation to the collection bag, and factors such as good social relations, leisure activities and absence of psychological problems and other health problems contributed for a better quality of life. Thus, the study concluded that surgery increases the concerns and profoundly impairs the patients’ quality of life in the first months^16^.

In another study performed in Brazil, stoma patients (n = 19) were characterized according to sociodemographic variables, identifying their needs. The results of the research indicated that patients were at risk of developing complications secondary to colostomy if they did not have support from health professionals of different specialties who worked in an integrated and multiprofessional way^17^.

Another study, also from Brazil, with a qualitative approach, analyzed the perception of ten patients with colostomy in relation to the use of the collection bag. The results showed that the life with the colostomy bag raises conflicting feelings, concerns and difficulties in dealing with the new situation^3^.

In a study carried out in Cuba, the adaptation was compared between patients of two groups: ileostomy and sigmoidostomy patients. It was observed that patients with ileostomy had a faster return to social life, with less psychological damage, unlike those with colostomy, who were more emotionally shaken, and who perceived rejection of their family or sexual partner. Thus, during the surgical planning, professionals should include the technical alternatives that favor the adaptation of the patient^18^.

In a Brazilian research, sociodemographic and clinical factors of patients with permanent intestinal stoma secondary to colorectal cancer were identified, correlating them with Quality of Life (QoL). The most affected components of the QoL were: *psychological* domain, with emphasis on women, individuals with lower income and individuals who had not received guidance on the stoma; *social* domain, especially in patients who did not have a sexual partner and who had metastasis; and, finally, the *physical* domain, especially in patients who had not received guidance before surgery on the stoma and those who did not have a sexual partner^19^.

In a study developed in Taiwan, the relationships between sociodemographic and clinical characteristics, spiritual well-being and psychosocial adjustment were examined in patients with colorectal cancer and colostomy. Spiritual well-being was significantly associated with psychosocial adjustment. Four predictors (change in income after surgery, disease severity, time elapsed after surgery, and spiritual well-being) accounted for 53% of the variation observed in the psychosocial adjustment^20^.

Another study carried out in China evaluated the impact of knowledge on the oesophageal and self-care capacity in the psychosocial adjustment of 54 permanent colostomy patients. The results showed that colostomy can negatively affect the social life of the patients, and psychosocial adjustment is correlated with the quality of life and the patient’s knowledge about the stoma. Patients with higher levels of knowledge about care achieved better adaptation when compared to people with less knowledge and greater dependence on other people to perform care^21^.

In another study conducted in Australia, the *Common Sense* model was used to assess disease perceptions, self-efficacy and coping strategies with the stoma, and to attempt to explain the symptoms of anxiety and depression among patients with faecal stoma. Perceptions of the disease, from moments prior to surgery until the creation of the stoma and the post-surgical adaptation, influenced the coping strategies adopted by the patients. The results confirmed the need to plan psychological interventions to understand the individual perceptions about the disease, instead of focusing exclusively on the coping modes used by patients with stoma^22^.

In another study, Chinese patients with colostomy were evaluated and the relationships between adaptation, self-care ability, and social support were described. The results indicated a positive correlation between self-care ability and social support, linked to the adaptation to the stoma. On the other hand, concerns about the odor and possible rejection that the stoma might induce in other people significantly contributed to poorer adjustment to the stoma^23^.

In China, another study investigated the quality of life, access to knowledge and needs for colostomy self-care in 142 colorectal cancer patients, and concluded that the teaching of self-care, with clear and objective language and methods, favors learning and, consequently, quality of life^24^.

Four primary articles were categorized in the thematic axis “effects of different intervention strategies to optimize psychosocial adjustment”.

The effects of a intervention in Chinese patients with permanent colostomy who attended a support program were evaluated. In this study, it was shown that social support is fundamental for a better adaptation of patients with permanent colostomy and that participation in a program for people with stoma provides a support network that promotes significant improvement in knowledge, self-efficacy, self-management and psychosocial adjustment to the colostomy^25^.

Another study carried out in China evaluated the effect of telephone follow-up by the stoma therapist nurse and the adjustment levels of patients with colostomy who had been discharged from hospital. Follow-up through telephone calls after hospital discharge was effective in improving satisfaction with care, reducing the complications in the colostomy, improving self-care skills, and increasing patient self-confidence to cope with the colostomy. Although performed at a distance, follow-up became an extremely important factor for better adaptation to the colostomy bag and, consequently, for the achievement of social adjustment^26^.

In another study, also conducted in China, the effectiveness of a stress management program in stoma patients was tested. The postoperative period can trigger symptoms of stress, anxiety and depression, because it is a transition period that requires adjustment to the new physical condition. After the participation in the program, satisfactory results of reduction of stress, depression and anxiety in the stoma patients were evidenced^27^.

The patients’ response to a type of supportive intervention was tested in another study, with some similar characteristics to the model evaluated in another research conducted in China^25^. In this study, carried out in Turkey, the effects of group interaction on the social adjustment of people with intestinal stoma were investigated. The interaction with other stoma patients was important, since group meetings and the exchange of experiences with other people who share similar situations favors better adaptation to the stoma^28^.

Five primary articles were included in the thematic axis “understanding of the subjective experience of disease/becoming ill”, as described below.

In a Scottish study, the correlation between the changes brought about by the colostomy bag and the psychological aspects of patients in a sample of 86 people during the one to four months after surgery was evaluated. Half of the participants reported the sensation of having lost control of their own body and therefore avoided social and leisure activities, and the odor led them to social isolation. Specialized assistance from a stoma therapist, along with psychological support, helped in the rehabilitation of these patients^29^.

The objective of the study^30^ conducted in Sweden was to describe the experience of women living with colostomy after rectal cancer surgery. The results of the research indicated that the diagnosis of cancer prompt recurrent thoughts of life and death, but living with the colostomy meant a “victory” and a “relief” for surviving the stigmatized disease and the penalty of death. Thus, surgery for tumor resection was resignified as a saving resource. This feeling favored a better adaptation to the colostomy bag, with greater consideration and adherence to the guidelines provided by the nursing team^30^.

Another study developed in Brazil aimed at understanding the experiences of people with stoma resulting from cancer, with the guiding question “what has changed in your life after the surgery, with the creation of the stoma?”. In the interpretation of the discourses some convergent feelings emerged, which revealed the existential theme: *the temporality of existing in the stoma world*. The analysis revealed that colostomy following colorectal cancer imposes important physical, emotional and social changes on the patients, with the need to transcend the restrictions imposed by the disease to adapt to the use of the colostomy bag and resume activities of daily living^31^.

The strategies developed by the patients to deal with the stoma were described in a research carried out in Spain. The content analysis of the interviews revealed three categories, around which the different strategies were developed: self-care, adaptation to corporal change and self-help. The researchers concluded that discovering the strategies used may be fundamental for nursing professionals to offer high-quality care, focused on the real needs of the patients during the process of adaptation to the stoma^32^.

In Ireland, a research was carried out in order to understand how stomized patients perceived their life and the main issues faced, getting to the conclusion that stoma patients have impaired mental health as well as sexual dysfunctions when compared to the general population. To mitigate these symptoms, it was recommended to share experiences with other stoma persons and receive a follow-up assistace with a stoma therapist to avoid complications with the stoma^33^.

The research carried out in Spain had the objective to verify the occurrence of difference in relation to QoL among individuals with temporary and permanent stoma. The results did not show statistically significant differences in the QoL of these patients. It is relevant to consider that the QoL construct is defined as the subjective perception of the individuals about their position in the world and, as such, reflects the subjects’ own appreciation of their life. It can be inferred from this study that the perspective of living for the rest of the life with the stoma is not a factor that negatively impacts the self-assessment, at least in the first three postoperative months^34^.

In a study in Turkey with 14 patients who had been stomized two months ago, the authors described their experiences with sexual function and their perceptions and expectations about stoma therapist nurses. It was observed that people with stoma experienced changes in body image, with decreased sexual desire, avoiding intercourse and abstained from sleeping with their partners. Male respondents described erectile dysfunction and those interviewees reported dyspareunia. Participants reported the need to receive more information from stoma therapist nurses on sexuality and post-stoma challenges^35^.

## Discussion

The evidence on the psychological aspects of stomized patients during surgical treatment is scarce in the national and international literature, especially in relation to the preoperative period, which involves physical and emotional preparation for surgery, and the postoperative period, with physiological stabilization, specialized assistance and preparation for discharge.

The psychological demand, through the analysis of this sample, showed that the need to live 24 hours a day connected to a colostomy bag arouses negative feelings, impacting all aspects of the patient’s life. These changes may or may not be irreversible, depending on the clinical condition of each patient, professional support, family support and the use of coping strategies ^3^ ^,^ ^17^ ^,^ ^21^.

In order to achieve rehabilitation, specialized care should be interdisciplinary, including perioperative education, reception with professional support and individualized therapy, in order to promote a more satisfactory acceptance of the new condition^35^. The use of coping strategies by the patients attenuate the impact of the illness and improve their psychological well-being.

Despite consistent results on the repercussions for stomized patients, there is a gap in studies focusing on the psychological impact during hospital stay resulting from the surgical treatment with stomization. This fact can be considered a limitation for the scope of the complete analysis of the aspects that characterize the psychological dimension of the patients in this moment of crisis.

Stoma patients presented worse quality of life in the first months post-surgery when compared to the six month. This illustrates that adaptation and acceptance require time and interdisciplinary care, encompassing psychological aspects, stoma care and the collecting bag, with prevention of complications, and support to cope with the stoma^16^ ^,^ ^24^ ^,^ ^29^.

Assistance for this clientele should be planned considering the physiological aspects along with psychological care, aiming at integral care of the patient’ needs.It is essential that all professionals involved participate effectively in the care process, characterized as continuous follow-up during hospitalization for surgical treatment.

In this perspective, the results in this review confirmed the need to plan pre- and postoperative psychological interventions, for the preparation until the adaptation to the stoma. This makes it possible to know the individual perceptions about illness/becoming ill, rather than focusing exclusively on coping strategies that patients use after the surgical procedure^22^.

The results also showed that negative feelings, such as anxiety, depression and anguish, arise concomitantly with concerns about social life and insecurity by reintegration of previous social roles and functions. Thus, health professionals should recognize and assist/encourage patients in their efforts to reduce such concerns by providing professional support for the development of instrumental, expressive, and social skills.The ability to perform the care of the stoma and of the skin around the stoma, the competence to identify problems and complications, and the search for appropriate physical and psychosocial solutions should be primarily stimulated^21^ ^,^ ^25^ ^,^ ^30^.

Strategies of group interaction among patients experiencing the same problem can be used in clinical practice, mainly for greater proximity and approach to psychosocial issues. This recommendation was seen in several studies, showing the importance of specialized follow-up after hospital discharge, with emphasis on self-care, acceptance of emotional needs to ease the barriers and difficulties in resuming daily life^(3,17, 31)^.

There is also a need for reflection on the organization of the health system to include adequate care for patients with stoma in order to integrate them into society as citizens and to include new demands for care. For this to occur, it is not enough to recognize only the changes related to the physical and corporal dimension; it is necessary that the health professionals offer support for the inclusion of these patients in society. Adaptation after hospital discharge may be favored by effective coping with embarrassment situations, with the understanding of their anguish, fears and doubts during the surgical treatment, since the physical and psychoemotional preparation until the adaptation to the changes caused by the stome in their life. The care from health professionals should go much further than providing *kits*, booklets and self-care guidance regarding the colostomy and the collection bag. Expanding the possibilities of active social life despite the need for adaptations is also important. In addition, spaces for discussion of social prejudice and stigmas can be disseminated in society for the implementation of integral care^31^.

Most of these studies were performed with ambulatory patient in the postoperativeperiod. However, the results that make up this review point to the importance of perioperative follow-up, because it is in this moment that the doubts about the surgery and its consequences should be clarified, as well as self-care to minimize fears and anxieties during surgical treatment and in prevention of future complications^19^ ^,^ ^21^ ^,^ ^25^. Consequently, there is a need for future studies addressing the psychological aspects during surgical treatment, for scientific evidence on the importance of interdisciplinary care.

The results of this analysis point to the need to improve the care for stomized patients during the hospital stay, through a protocol for psychological follow-up, as a contribution of psychology to the interdisciplinary team.

## Conclusion

With this review, it was possible to identify a shortage of scientific productions on stomized patients during hospitalization in the pre and post-operative period; no study directly contemplated this period, but rather the period after hospital discharge, with ambulatory patients under follow-up. Therefore, gaps in the production of knowledge about the psychological aspects related to surgical treatment and interdisciplinary care were identified.

In some studies, the authors acknowledged the importance of the preoperative approach of the patients, including psychological aspects, to reduce the possibility of postoperative complications and contribute to the patients’ adaptation and coping with the stoma and the physical and psychosocial rehabilitation.

The planning of perioperative care should include reception and instructions about the surgery and its consequences, with the insertion and involvement of family members, as well as enable the effective participation of the patients in decision making in clinical situations. In this planning, the need to include emotional, social, cultural and spiritual aspects stands out.
